# Supplementary material for: Mapping the serum proteome to neurological diseases using whole genome sequencing
Source: Nat Commun. 2021 Dec 2;12:7042. doi: 10.1038/s41467-021-27387-1 (PMC8640022; doi:10.1038/s41467-021-27387-1)
Supplement: Supplementary file 1 — Supplementary Information [file 41467_2021_27387_MOESM1_ESM.pdf]

Supplementary Figures

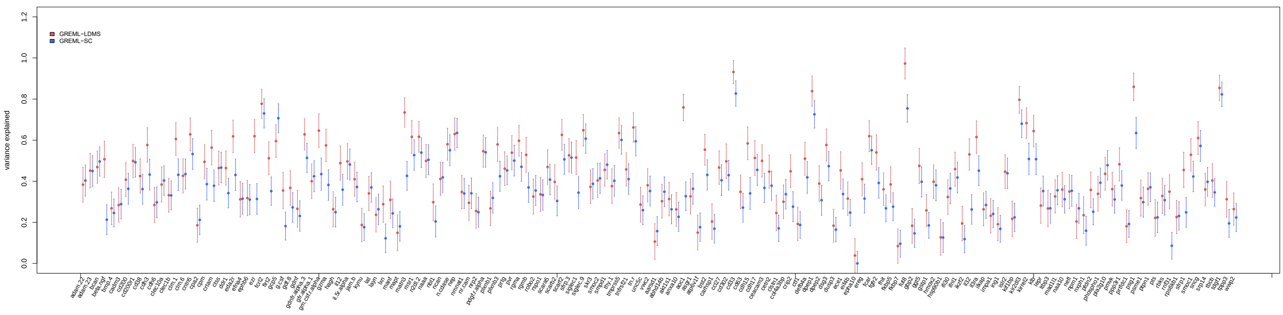

Supplementary Figure 1. Comparison of of h^2 values for each analysed protein in MANOLIS, produced by single component GREML (GREML-SC) versus multi component GREML (GREML-LDMS). n=1,356 MANOLIS samples. GREML-LDMS was run using four GRMs, each generated using variants that have been stratified into one of four quartiles (total 25,371,797 variants) according to their LD scores (see Methods). Data are represented as mean variance explained +/- SEM.

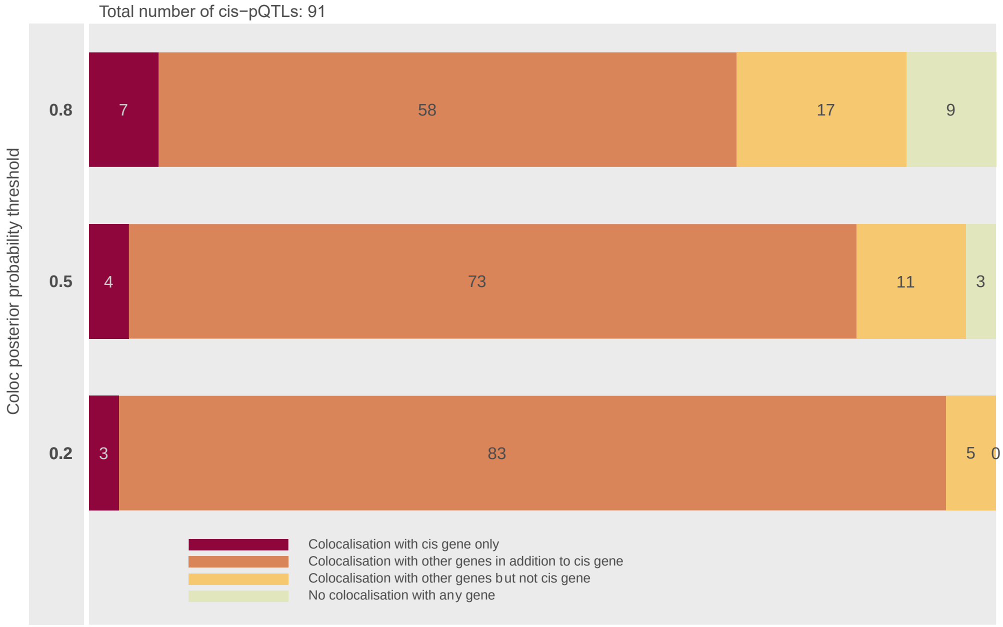

Supplementary Figure 2. Colocalisation analysis of cis-pQTLs with eQTLs from adjacent genes within 2Mb. This analysis was performed to determine the sensitivity and specificity of using eQTL colocalisation analysis to map trans-pQTLs to their causal genes. The numbers within each coloured rectangle represent the number of cis-pQTLs that correspond to the legend provided. Positive colocalisation was defined according to three different posterior probability thresholds (y-axis). The cis gene refers to the gene encoding the analysed protein.

Supplementary Notes

Supplementary Note 1

Sensitivity and specificity of using eQTL colocalisation to map *trans*-pQTLs

Colocalisation analysis of *trans*-pQTLs and nearby gene expression QTLs (eQTLs) can be a useful tool for mapping *trans*-pQTLs to their causal genes. To determine the accuracy of this method, we performed colocalisation analysis for all *cis*-acting pQTLs with the expression of all genes in all available tissues (GTEx) within 2Mb of the independent variant. A median of 22 genes were analysed for each *cis*-pQTL. Using a threshold of CLPP4 (Methods)>0.8 to define positive colocalisation, 65 of 91 *cis*-pQTLs colocalised with expression of the protein-encoding gene, giving the method a sensitivity of 71.4% (Supplementary Figure 2). Specificity, however, was very low, with only seven pQTLs (7.7%) colocalising with just the *cis* gene. Relaxing the threshold to CLPP4>0.5 and CLPP4>0.2 increased sensitivity markedly (84.6%; 94.5%), but also reduced specificity (4.4%; 3.3%) (Supplementary Figure 2). Therefore, while gene expression QTL colocalisation method is sensitive enough to capture the causal gene, it should be used in complement with other tools and resources, such as literature mining, protein-protein interaction (PPI) databases, and existing experimental evidence.

Supplementary Note 2

CD33 as a susceptibility marker for Alzheimer's disease

Through causal inference analysis, we identified serum CD33 as a potential susceptibility marker for Alzheimer's disease (AD), further supported by the high heritability of serum CD33 (88%). Following up on this observation, we tested for colocalisation between serum CD33 and family history of AD (FHAD) using publicly available summary statistics from a large GWAS by Marioni et al.[6] We observed, however, no evidence of positive colocalisation for all three causal variants (CLPP4 < 7.4%), likely owing to a lack of association of the locus to FHAD (CLPP1 > 90%).

Supplementary Note 3

Drug target evaluation

Among the proteins identified as targets of drugs that are approved or in the later stages of clinical trials, several are causal for diseases different from the drug's original indication. In addition to the potential repurposing of CD33-targeting drugs for Alzheimer's disease treatment, we also find evidence of colocalisation between decreased serum leptin receptor (LEPR) and

migraine; LEPR is a target for leptin and metreleptin, which are both used for the treatment of obesity and dyslipidemias where there is a deficiency of LEP and LEPR. While the evidence surrounding leptin's role in migraine pathology is conflicting in existing literature, this highlights the possibility of using leptin or metreleptin to treat leptin-deficient subtypes of migraine.

We also identify drugs that may not be appropriate for repurposing. For instance, a GPNMB-targeting drug, glembatumumab vedotin, is in clinical trials for the treatment of melanoma and breast cancer. We find evidence that GPNMB expression is increased in individuals with PD; however, as the cytotoxic drug does not cause changes in GPNMB levels, it is not recommended for PD treatment[1]. This example serves to illustrate the importance of ensuring the congruency of a drug's mechanism of action with disease pathophysiology.

Supplementary References

1. Storm, C. S. *et al. Finding drug targeting mechanisms with genetic evidence for Parkinson's disease*. http://biorxiv.org/lookup/doi/10.1101/2020.07.24.208975 (2020) doi:10.1101/2020.07.24.208975.
2. Giambartolomei, C. *et al.* Bayesian test for colocalisation between pairs of genetic association studies using summary statistics. *PLoS Genet.* **10**, e1004383 (2014).
3. Hemani, G. *et al.* The MR-Base platform supports systematic causal inference across the human phenome. *eLife* **7**, e34408 (2018).
4. Hemani, G., Tilling, K. & Davey Smith, G. Orienting the causal relationship between imprecisely measured traits using GWAS summary data. *PLOS Genet.* **13**, e1007081 (2017).
5. Hemani, G., Bowden, J. & Davey Smith, G. Evaluating the potential role of pleiotropy in Mendelian randomization studies. *Hum. Mol. Genet.* **27**, R195–R208 (2018).
6. Marioni, R. E. *et al.* GWAS on family history of Alzheimer's disease. *Transl. Psychiatry* **8**, 99 (2018).
