# Supplementary material for: Mapping the serum proteome to neurological diseases using whole genome sequencing
Source: Nat Commun. 2021 Dec 2;12:7042. doi: 10.1038/s41467-021-27387-1 (PMC8640022; doi:10.1038/s41467-021-27387-1)
Supplement: Supplementary file 3 — Description of Additional Supplementary Files [file 41467_2021_27387_MOESM3_ESM.pdf]

Description of Additional Supplementary Files

File Name: Supplementary Data 1
Description: Independent protein QTL variants of serum proteins comprising the Olink Neurology and Neuro-exploratory panels. In addition to results of the meta-analysis, percohort summary statistics are also included in the table. Independent cohort analysis was carried out using the linear mixed model implemented in GEMMA, and meta-analysis using the inverse variance-based method implemented in METAL. Reported p-values are not adjusted. Additional information includes the gene that each variant is mapped to, or for intergenic variants, the nearest protein-coding gene. Results from the novelty analysis are also indicated in the "novelty" column: "novel_protein" denotes proteins being analysed for pQTLs for the first time; "novel_signal" denotes novel pQTL loci for proteins that have previously been analysed; "novel_variant" denotes novel independent variants from known pQTL loci.

File Name: Supplementary Data 2
Description: Protein QTL studies referenced for novelty analysis. *The table in the link contains a comprehensive list of pQTL studies, which we referenced.

File Name: Supplementary Data 3
Description: Protein QTL variants with potential protein-altering consequences.

File Name: Supplementary Data 4
Description: Results of colocalisation analysis between pQTLs and eQTLs. Sheet 1: Results of colocalisation analysis between serum protein levels and expression of their encoding genes (eQTL data from GTEx), using independent cis-acting variants as the causal variant for each test. The table includes all results with colocalisation posterior probability (CLPP4) > 0.5. Sheet 2: Results of colocalisation analysis for trans-acting pQTLs. Colocalisation was tested between serum protein levels and expression of any gene within 2Mb of the causal variant. The table includes protein-gene pairs with CLPP4 > 0.5.

File Name: Supplementary Data 5
Description: Neurological phenomewide colocalisation results, with colocalisation posterior probability (CLPP4) > 0.5. Sheet 2: Genome-wide association studies used for colocalisation analysis.

File Name: Supplementary Data 6
Description: Causal protein-disease associations identified using two-sample Mendelian randomisation. „nsnp‟ represents the number of available SNPs included in the test; „method‟ refers to the statistical test used. Pvalues were corrected for multiple testing by controlling for false discovery rate (FDR) using the Benjamini-Hochberg method. Adjusted Pvalues are given in the column „p.BH‟. Sheet 1: Significant protein-disease associations from two-sample Mendelian randomisation analysis. Analysis excluded all pleiotropic loci, but included trans-acting loci. Sheet 2: Significant protein-disease associations from two-Sample Mendelian randomisation analysis, excluding pleiotropic loci and trans-acting loci. Sheet 3: Outcome IDs and names, as in MRBase. Manually downloaded summary statistics were also included, and are indicated in the table.

File Name: Supplementary Data 7
Description: Drug target evaluation. Sheet 1: Proteins that act as targets of approved drugs, according to the Drugbank database. Sheet 2: Proteins that act as targets for drugs that have at least completed Phase II of clinical trials, according to the OpenTargets platform. Proteins with pQTLs reported in this study are also denoted in the column „pQTL‟.

File Name: Supplementary Data 8
Description: List of proteins included in analysis.

File Name: Supplementary Data 9
Description: GCTA GREML metaanalysis for 184 serum proteins in the MANOLIS and Pomak cohorts. For multicomponent GREML (greml_ldms), NA values are given where REML analysis failed to converge in at least one cohort, likely owing to inadequate sample size.

File Name: Supplementary Data 10
Description: GWAS Catalog accession codes for access to summary statistics.
