# Supplementary material for: Mapping the serum proteome to neurological diseases using whole genome sequencing
Source: Nat Commun. 2021 Dec 2;12:7042. doi: 10.1038/s41467-021-27387-1 (PMC8640022; doi:10.1038/s41467-021-27387-1)
Supplement: Supplementary file 14 — Reporting Summary [file 41467_2021_27387_MOESM14_ESM.pdf]

Corresponding author(s): Eleftheria Zeggini

Last updated by author(s): Oct 27, 2021

Reporting Summary

Nature Portfolio wishes to improve the reproducibility of the work that we publish. This form provides structure for consistency and transparency in reporting. For further information on Nature Portfolio policies, see our Editorial Policies and the Editorial Policy Checklist.

Statistics

For all statistical analyses, confirm that the following items are present in the figure legend, table legend, main text, or Methods section.

n/a	Confirmed	
☐	☒	The exact sample size (*n*) for each experimental group/condition, given as a discrete number and unit of measurement
☐	☒	A statement on whether measurements were taken from distinct samples or whether the same sample was measured repeatedly
☐	☒	The statistical test(s) used AND whether they are one- or two-sided<br>*Only common tests should be described solely by name; describe more complex techniques in the Methods section.*
☐	☒	A description of all covariates tested
☐	☒	A description of any assumptions or corrections, such as tests of normality and adjustment for multiple comparisons
☐	☒	A full description of the statistical parameters including central tendency (e.g. means) or other basic estimates (e.g. regression coefficient) AND variation (e.g. standard deviation) or associated estimates of uncertainty (e.g. confidence intervals)
☐	☒	For null hypothesis testing, the test statistic (e.g. *F*, *t*, *r*) with confidence intervals, effect sizes, degrees of freedom and *P* value noted<br>*Give P values as exact values whenever suitable.*
☒	☐	For Bayesian analysis, information on the choice of priors and Markov chain Monte Carlo settings
☒	☐	For hierarchical and complex designs, identification of the appropriate level for tests and full reporting of outcomes
☐	☒	Estimates of effect sizes (e.g. Cohen's *d*, Pearson's *r*), indicating how they were calculated

Our web collection on statistics for biologists contains articles on many of the points above.

Software and code

Policy information about availability of computer code

Data collection	No software was used for data collection.
Data analysis	The sequencing data was processed using Illumina2BAM 6638a24 and aligned with BWA MEM 0.7.8 to the hg38 reference (GRCh38) with decoys (HS38DH). Aligned reads were processed and called using GATK v3.5-0-g36282e4. For the association analysis and identification of independent pQTLs, the following public software versions were used: GEMMA v.0.94, METAL (released 2011-03-25), Peakplotter (commit cd2c6d325843dbbe5091aeffe9d0589ec31a35d6; https://github.com/hmgu-itg/peakplotter), Plink 1.9 and GCTA 1.92. Statistical analysis was performed using R v.4.0.3. For two-sample Mendelian randomisation, we used the R package TwoSampleMR v0.5.6; and for colocalisation, the gtx R package (commit 9afa9597a51d0ff44536bc5c8eddd901ab3e867c; https://github.com/tobyjohnson/gtx/). Heritability analysis was carried out using the --reml function in GCTA 1.92. All additional scripts developed as part of the study are available at https://github.com/hmgu-itg/ .

For manuscripts utilizing custom algorithms or software that are central to the research but not yet described in published literature, software must be made available to editors and reviewers. We strongly encourage code deposition in a community repository (e.g. GitHub). See the Nature Portfolio guidelines for submitting code & software for further information.

Data

Policy information about availability of data

All manuscripts must include a data availability statement. This statement should provide the following information, where applicable:

- Accession codes, unique identifiers, or web links for publicly available datasets
- A description of any restrictions on data availability
- For clinical datasets or third party data, please ensure that the statement adheres to our policy

The MANOLIS sequencing data used in this study is available at the European Genome-Phenome Archive (EGA) under accession number EGAS00001001207 [https://ega-archive.org/studies/EGAS00001001207]. The Pomak sequencing data have not been deposited to the EGA as the data and the information derived from it are culturally and politically sensitive in the context of this religiously isolated population. We will consider requests to access the data by researchers when an alternative cohort cannot reasonably be used for their research, and will respond to such requests within six months. Summary statistics generated in this study are available for download in the GWAS Catalog. Accession codes and the respective hyperlinks are provided in Supplementary Data 10.

Field-specific reporting

Please select the one below that is the best fit for your research. If you are not sure, read the appropriate sections before making your selection.

☒ Life sciences ☐ Behavioural & social sciences ☐ Ecological, evolutionary & environmental sciences

For a reference copy of the document with all sections, see nature.com/documents/nr-reporting-summary-flat.pdf

Life sciences study design

All studies must disclose on these points even when the disclosure is negative.

Sample size	Sample sizes were determined so as to provide 80% power to detect single-variant associations of medium to large effect sizes down to allele frequencies of 1%.
Data exclusions	25 samples in MANOLIS and 31 samples in Pomak were excluded from each discovery cohort due to contamination, duplication or sex mismatches. A further 52 and 37 MANOLIS samples, and 68 and 60 Pomak samples failed vendor QC for the Neurology and Neuro-exploratory panels, respectively, and were excluded from analysis. None of the measured serum proteins were excluded.
Replication	Replication in an independent cohort other than MANOLIS and Pomak was not carried out. Rather, we applied stringent criteria in order to consider a variant to be significantly associated with a protein trait: variants must (i) have a p-value below the study-wide significance threshold (P<1.05e-10); (ii) be nominally significant (P<1e-4) in both MANOLIS and Pomak; (iii) have the same direction of effect. We also replicate previously published pQTLs (a list of studies can be found in Supplementary Data 2); 75 of 214 (35%) pQTLs replicate directly, while an additional 50 (23%) pQTLs replicate after conditioning on previous pQTLs (see 'Novelty' in Methods).
Randomization	As this was a population cohort, samples were not divided into groups. A relatedness matrix was used in the analysis to correct for non-independence of samples in each cohort.
Blinding	Blinding was not relevant as this is an observational study where samples were not grouped by intervention.

Reporting for specific materials, systems and methods

We require information from authors about some types of materials, experimental systems and methods used in many studies. Here, indicate whether each material, system or method listed is relevant to your study. If you are not sure if a list item applies to your research, read the appropriate section before selecting a response.

Materials & experimental systems

n/a	Involved in the study	
☒	☐	Antibodies
☒	☐	Eukaryotic cell lines
☒	☐	Palaeontology and archaeology
☒	☐	Animals and other organisms
☐	☒	Human research participants
☒	☐	Clinical data
☒	☐	Dual use research of concern

Methods

n/a	Involved in the study	
☒	☐	ChIP-seq
☒	☐	Flow cytometry
☒	☐	MRI-based neuroimaging

Human research participants

Policy information about studies involving human research participants

Population characteristics	Both MANOLIS and Pomak are population isolates with high levels of relatedness, exhibiting genetic characteristics including extensive haplotype sharing and the enrichment of missense and rare variants. In MANOLIS, the mean age is 61 (N=1,457; SD=19) overall, 63 in females (N=789; SD=18) and 59 in males (N=618; SD=20). In Pomak, mean age is 45 (N=1,605; SD=15) overall, 43 (N=1,124; SD=15) in females and 49 (N=481; SD=16) in males. Relatedness was corrected for using a genetic relatedness matrix in the association analysis.
Recruitment	Subjects were recruited at local clinics on a voluntary basis. The population characteristics described above reflect this. To control for demographic and geographic bias, sex, age, and population structure (represented by a genetic relatedness matrix) were used as covariates in the analysis. As this study is a cross-sectional population cohort without stratification or subgroups, recruitment bias is unlikely to affect the results.
Ethics oversight	The study was approved by the Institutional Review Board of Harokopio University and the Greek Ministry of Education, Lifelong Learning and Religious Affairs. The MANOLIS and Pomak studies were approved by the Harokopio University Bioethics Committee and informed consent was obtained from every participant.

Note that full information on the approval of the study protocol must also be provided in the manuscript.
